# Factors associated with timely initiation of antenatal care among reproductive age women in The Gambia: a multilevel fixed effects analysis

**DOI:** 10.1186/s13690-024-01247-y

**Published:** 2024-05-17

**Authors:** Silas Selorm Daniels-Donkor, Agani Afaya, Dennis Bomansang Daliri, Timothy Tienbia Laari, Solomon Mohammed Salia, Mabel Apaanye Avane, Richard Adongo Afaya, Vida Nyagre Yakong, Martin Amogre Ayanore, Robert Kaba Alhassan

**Affiliations:** 1 School of Health Sciences, University of Dundee, Dundee, Scotland, UK; 2 Department of Nursing, School of Nursing and Midwifery, University of Health and Allied Sciences, Ho, Ghana; 3 Department of Global Health, School of Public Health, University for Development Studies, Tamale, Ghana; 4 Presbyterian Primary Health Care (PPHC), Bolgatanga, Ghana; 5 Department of Preventive Health Nursing, School of Nursing and Midwifery, University for Development Studies, Tamale, Ghana; 6 Department of Midwifery and Women’s Health, School of Nursing and Midwifery, University for Development Studies, Tamale, Ghana; 7 Department of Health Policy Planning and Management, Fred N. Binka School of Public Health, University of Health and Allied Sciences, Ho, Ghana; 8 Centre for Health Policy and Implementation Research, Institute of Health Research, University of Health and Allied Sciences, Ho, Ghana; 9 University of Dundee, Scotland United Kingdom, Dundee, Scotland, UK

**Keywords:** Antenatal care, Reproductive-age women, GDHS, The Gambia

## Abstract

**Background:**

A significant factor impacting the incidence of maternal and neonatal fatalities is the timely initiation of antenatal care (ANC) services in healthcare facilities. Despite the recommendations by the World Health Organization and the numerous benefits of timely initiation of ANC, studies have revealed that the overall prevalence of timely ANC initiation in 36 sub-Saharan African countries remains low and women in The Gambia also initiate ANC late. However, no known study in The Gambia has focused on assessing the factors associated with timely initiation of ANC at the time of writing this paper. Thus, this study aimed to assess the prevalence and factors associated with the timely initiation of ANC among reproductive-age women in The Gambia.

**Methods:**

A cross-sectional survey design was used in this study and conducted among 5,734 reproductive-age women using data from the 2019–2020 Gambia Demographic and Health Survey (GDHS). Using STATA version 14.0, we conducted the analysis using descriptive and inferential statistics. Multilevel logistic regression models were fitted to determine the factors associated with timely ANC utilization and adjusted odds ratios were used to present the results with statistical significance set at *p* < 0.05.

**Results:**

The overall prevalence of timely initiation of ANC services among reproductive-age women in The Gambia was 43.0%. We found that women aged 30–34 [aOR = 1.79, 95% CI = 1.30–2.47], those who were married [aOR = 2.69, 95% CI = 1.85–3.90] as well as women from the richest households [aOR = 1.63, 95% CI = 1.20, 2.20] had higher odds of seeking timely ANC services as compared to their counterparts. Also, those who had given birth to two children [aOR = 0.74, 95% CI = 0.6 -0.91] had lower odds of initiating timely ANC as compared to those who had given birth only once. Women who reside in rural areas [aOR = 1.72, 95%CI = 1.34, 2.20] also had higher odds of seeking timely ANC services than those residing in urban areas.

**Conclusion:**

Individual-level factors such as maternal age, marital status, parity, wealth status, place of residence, and religion were associated with the timely initiation of ANC services among reproductive-age women. These factors ought to be considered in efforts to increase the timely initiation of ANC among reproductive-age women in The Gambia.

**Table:** 

Text box 1. Contributions to the literature
• There is limited evidence on factors associated with timely initiation of antenatal care (ANC) among women of reproductive age in The Gambia using nationally representative data.
• The study identified a low prevalence of timely initiation of ANC among reproductive age women. Factors such as women aged 30–34 years, those who were married, women from the richest households, and those residing in urban areas were associated with timely ANC initiation.
• Policies and interventions targeted to improve timely ANC initiation among reproductive age women could consider these factors when designing them.

## What is known

The Gambia, a low-income nation in West Africa, has made some attempts over the years to enhance maternal, neonatal, and child health, with 81.7% of expectant mothers giving birth in medical facilities and 82.7% receiving professional care. However, rates of mortality for children under five and mothers are still high, estimated at 54 per 1,000 in 2013 and 706 per 100,000 live births in 2015, respectively. One strategy for reducing maternal mortality is utilizing ANC during pregnancy. The incidence of timely ANC initiation was found to be 38% in 36 SSA countries, despite the WHO's recommendations and the multiple advantages of prompt ANC. Studies have confirmed that pregnant women in The Gambia are mostly satisfied with the ANC services they receive in both public and private facilities, however, they initiate ANC attendance late.

## What does the study add

To the best of our knowledge, no study has been conducted in The Gambia to determine the prevalence and factors associated with the timely initiation of ANC using a nationally representative data. The overall prevalence of timely initiation of ANC services among reproductive-age women in The Gambia was 43.0%. Our study revealed that women aged 30–34, those who were married as well as women from the richest households had higher odds of seeking ANC services early. Also, those who gave birth to two children had lower odds of initiating timely ANC as compared to those who have given birth only once. Women who reside in rural areas also had higher odds of seeking timely ANC services than those residing in urban areas.

## What are the implications for clinical practice, public health and/or research

When developing new policies or evaluating existing ones regarding early ANC uptake, factors such as maternal age, parity, wealth status, place of residence, and region should be taken into account to help promote early ANC attendance, which will aid in the decrease of maternal mortality in The Gambia. Clinicians and public health practitioners could consider the above findings when developing interventions to promote early initiation of ANC and when providing public health education among women on the importance of early ANC.

## Background

Despite the reduction of Maternal Mortality Rates (MMRs) globally by 38% between the years 2000 and 2017 [1], there has not been a significant reduction in low- and middle-income countries (LMICs) as preventable neonatal, maternal, and childhood deaths remain high in these countries accounting for 94% of the total maternal deaths that occur every day [2]. Over the years, sub–Saharan Africa (SSA) has remained disadvantaged recording 77% of these deaths [2]. Not only that, stillbirth has also remained a challenge in SSA even though there has been a global decline of 58.3% between the years 2000 and 2015 [3]. With the current trends, it will be difficult for these countries to attain the Sustainable Development Goals (SDGs) as maternal and neonatal mortality remains a key indicator in meeting this target [4]. Thus, these countries need to accelerate their progress in reducing MMR by at least 7.5% annually in order to achieve the SDG goal by 2030 [5].

The Gambia is one of the low-income countries in West Africa that has made some efforts to improve maternal, newborn, and child health over the years, with pregnant women delivering in health facilities estimated at 81.7% and those attended to by skilled professionals at 82.7% [6]. However, there remain high rates of under-five mortality and maternal mortality rates estimated at 54 per 1000 in 2013 and 706 deaths per 100 000 live births in 2015 respectively [7, 8]. Utilization of ANC services in health facilities, as well as skilled birth attendance and postnatal care, remain crucial in influencing the rates of maternal and neonatal deaths [9]. It has been recommended by the WHO for pregnant women to practice focused ANC for a minimum of four ANC visits with their first visit within the first 12 weeks of gestation [10]. Evidence has revealed that perinatal deaths were still on the increase even with the four ANC contacts [11]. The WHO therefore, in 2016, recommended a minimum of eight contacts with the health care provider with the goal of giving the opportunity for high-quality care, including medical care, assistance, and information that is current and pertinent during pregnancy where the initial contact occurs during the first 12 weeks of pregnancy, followed by contacts at 20, 26, 30, 34, 36, 38, and 40 week [11].

When pregnant women make their first early pregnancy care contact with a medical professional during the first trimester of their pregnancies, ANC is timely commenced [12]. Seeking ANC services early, timely, and regularly gives healthcare providers the opportunity to educate pregnant women on the variety of events that come with pregnancy and childbirth [11, 13]. Not only that, it also gives healthcare providers the opportunity to screen and treat health problems associated with pregnancy including infections [14]. Literature has revealed that, the risk of neonatal mortality will reduce by 39% in SSA should pregnant women utilize ANC services at least once and are attended to by a skilled health care provider [15].

Despite the recommendations by WHO and the numerous benefits of timely ANC, a study has revealed that the overall prevalence of timely ANC initiation in 36 SSA countries was 38% [16]. Evidence from several studies have revealed that maternal age, type of employment, maternal level of education, marital status, place of residence, parity, intention to get pregnant, mass media exposure, who makes a decision on accessing health care, cost of healthcare treatment and distance to health care facilities are factors associated with timely initiation of ANC [13, 17–19]. Studies have confirmed that pregnant women in The Gambia are mostly satisfied with the ANC services they receive in both public and private facilities accounting for satisfaction rates of 79.9% and 97.9% respectively [20]. This has resulted in high rates of ANC attendance among pregnant women in The Gambia with over 90% of them attending ANC at least once when pregnant [21]. Despite this high ANC attendance rate, they however initiate the attendance late. About 8.1% of the women utilized ANC within the first trimester of pregnancy, 62.8% initiated in the second trimester while 29.1% utilized ANC in the third trimester [21]. To the best of our knowledge, no study has been carried out to assess factors affecting timely ANC initiation in The Gambia. Therefore, this study sought to assess the prevalence and factors associated with timely initiation of ANC services among women of reproductive age in The Gambia using nationally representative data.

## Methods

### Data source

This study used the 2019–2020 Gambia Demographic and Health Survey (GDHS) report which is a national representative data that gathers data on gender, nutrition, HIV/AIDS awareness, and reported sexually transmitted infections (STIs), maternal and child health, including the use of ANC services, as well as fertility rates and preferences, contraceptive use, infant, child, and neonatal mortality rates, and maternal mortality. The data collection period for the GDHS was from 21st November 2019 to 30th March 2020. The survey was executed by The Gambia Bureau of Statistics (GBoS) in collaboration with the Ministry of Health (MoH). Technical assistance was provided by ICF-lnternational through the Demographic and Health Survey (DHS) Program which is a United States Agency for International Development (USAID)-funded program. Worldwide the USAID supports the implementation of population and health surveys. Substantial funding for the 2019–2020 GDHS was from the United Nations Population Fund (UNFPA), and other important organizations and agencies [22].

Administratively, there are eight Local Government Areas (LGAs) in The Gambia. The LGAs are subdivided into districts and the district are also subdivided into settlements which can form an enumeration area (EA). A two-stage stratified sampling was used to select the sample for the 2019–20 GDHS. The first stage involved the selection of 281 EAs with a probability proportional to their size within each sampling stratum. The second stage included the systematic sampling of the 25 households from each cluster with a developed sample frame resulting in a total sample size of 7,025 selected households. From the households 12,481 women aged 15–49 were identified for individual interviews and 11865 women completed the interviews yielding a response rate of 95%. The current study sampled 5, 734 reproductive age women for the analysis after data cleaning [22].

### Outcome variable

The outcome variable in our study was timely initiation of ANC services. According to the WHO, ANC is said to be timely initiated when pregnant women have their first ANC contact with the health care provider within the first trimester of pregnancy [11]. The respondents in this study were asked “timing for first antenatal check” in the 2019/2020 GDHS. We classified women who mentioned they had their first ANC checks “in the first three months” as “Timely ANC” and women who mentioned they had their first ANC “after three months” as “Late ANC”. We coded “Timely ANC” as “1” and “Late ANC” as “0”. Women who could not tell which month they first initiated ANC services were excluded from the study.

### Explanatory variables

The study took into consideration 16 independent variables after a careful review of their impact on timely ANC in other relevant studies that have been published in the sub region [13, 17–19].The variables were maternal age, marital status, educational status, occupation, religion, frequency of reading newspapers or magazine, frequency of listening to radio, frequency of watching television, sex of household head, wealth index, place of residence, parity, permission to go to the hospital, getting money for treatment, distance to health facility and region. Marital status was classified into “never married”, “married” and “widowed/divorced/separated”. Also, parity has been classified into “one birth”, “two births”, “three births” and “four or more births”. We classified occupation into “not working” and “working”. Religion was classified into “Islam” and “Christianity”.

### Statistical analysis

All statistical analysis was conducted using STATA version 14.0. The analysis was done using descriptive and inferential statistics. Frequencies and percentages were used to show the distribution of the study sample and to present the prevalence of timely initiation of ANC. Next, to the analysis of the association between the explanatory factors (individual and contextual) and the outcome variable (timely initiation of ANC), we fitted two-level multivariate multilevel logistic regression models. The two-level modelling implies that women were nested within households while the households were also nested within clusters. To cater for the unexplained variability at the contextual level, the clusters were considered as random effects. The analysis took four models. The first model (model O) was the random intercept without any explanatory variable. The second model (model I) was fitted using the individual-level variables against the timely initiation of ANC. The third model (model II) consisted of the contextual level factors while the complete model (model III) consisted of all the explanatory variables against timely initiation of ANC. The models comprised of fixed and random effects.

The outcome of the fixed effects analysis was presented as adjusted odds ratio (aOR) with their confidence intervals (CI). The random effects were assessed using Intra-Class Correlation (ICC). To compare the models, we used the log-likelihood ratio and the Akaike’s Information Criterion (AIC) test. A multicollinearity test between the independent variables was conducted using the Variance Inflation Factor (VIF) command and results indicated that none of the variables was above the threshold (Max VIF = 2.56, Min VIF = 1.05, Mean VIF = 1.49). We applied the inherent sample weight (v005/1000000) and the survey (svy) command in Stata to account for the complex sampling structure of the DHS data.

### Ethical approval

We sought and got approval from the MEASURE DHS to use the 2019–2020 GDHS data for this study. Ethical approval was sought from the institutional review boards at ICF and The Gambia Government/Medical Research Council Joint Ethics Committee before the commencement of data collection [22]. This survey followed the standard guidelines for conducting human research as stated in the declaration of Helsinki.

## Results

### Socio-demographic characteristics

Table 1 presents the distribution of respondents’ socio-demographic characteristics and the proportion of reproductive age women who timely initiated ANC services. A total sample of 5, 734 reproductive age women were included in the study. It was found that approximately 29% of the women were in the age group of 25–29. Majority of them (91.6%) were married with more than half (66.8%) residing in urban areas. Majority (97.3%) were followers of Islam, while males dominated (83.4%) as household heads. Almost half (45.8%) had no formal of education and approximately 22% of them were from the poorest households. More than half (66.6%) of them belong to the working group. Majority (96.3%) of the women had no problem with either having permission to attend hospital or distance to the health facility while 72.1% of them also do not have a problem getting money to the hospital. With regards to media exposure, majority of the women (91%) did not read newspaper at all, 36% also did not listen to radio at all while approximately more than half (51%) of them watch television at least once a week.

**Table 1:** Socio-demographic characteristics of respondents using GDHS 2019–2020

Variables	Weighted N	Weighted %	Timing of 1st antenatal check (months)		
			**Late ANC (%)**	**Timely ANC (%)**	***p*-value**
**Individual level**					
**Age (years)**					< 0.001
15–19	297	5.3	53.0	47.0	
20–24	1,018	17.7	53.4	46.6	
25–29	1,635	28.5	52.5	47.5	
30–34	1,216	21.2	45.3	54.7	
35–39	1,005	17.5	48.0	52.0	
40–44	431	7.5	47.6	52.4	
45–49	132	2.3	48.7	51.3	
**Marital status**					< 0.001
Never married	274	4.8	76.4	23.6	
Married	5,255	91.6	48.7	51.3	
Widowed/Divorced/Separated	205	3.6	56.9	43.1	
**Educational status**					< 0.001
No education	2,624	45.8	47.1	52.9	
Primary	1,008	17.6	49.0	51.0	
Secondary	1,831	31.9	56.2	43.8	
Higher	271	4.7	48.9	51.1	
**Occupation**					< 0.001
Not working	1,914	33.4	51.0	49.0	
Working	3820	66.6	49.5	50.5	
**Religion**					0.019
Islam	5,577	97.3	49.7	50.3	
Christianity	157	2.7	62.2	37.8	
**Frequency of reading newspaper or magazine**					0.372
Not at all	5,220	91.0	49.7	50.3	
Less than once a week	374	6.5	53.3	46.7	
At least once a week	140	2.5	53.7	46.3	
**Frequency of listening to radio**					0.109
Not at all	1,292	36.0	48.9	51.1	
Less than once a week	2,203	33.0	51.9	48.1	
At least once a week	2,239	31.0	48.9	51.1	
**Frequency of watching television**					< 0.001
Not at all	1,322	23.1	42.8	57.2	
Less than once a week	1,503	26.2	54.7	45.3	
At least once a week	2,909	50.7	49.9	50.1	
**Sex of household head**					0.170
Male	4,783	83.4	49.6	50.4	
Female	950	1.6	52.2	47.8	
**Parity**					< 0.001
1 birth	1,151	20.1	52.7	47.3	
2 births	971	16.9	55.1	44.9	
3 births	971	16.9	48.9	51.1	
4 or more births	2,641	46.1	47.5	52.5	
**Getting permission to go to the hospital**					0.519
Big problem	212	3.7	52.4	47.6	
Not a big problem	5,522	96.3	49.8	50.2	
**Getting money for treatment**					0.907
Big problem	1,599	27.9	49.0	51.0	
Not a big problem	4,135	72.1	49.9	50.1	
**Distance to health facility**					$<$ 0.001
Big problem	1,481	3.7	45.9	54.1	
Not a big problem	4,253	96.3	51.7	48.3	
**Household level**					
**Wealth index**					< 0.001
Poorest	1,237	21.6	41.3	58.7	
Poorer	1,202	21.0	50.0	50.0	
Middle	1,203	21.0	55.3	44.7	
Richer	1,091	19.0	59.4	40.6	
**Community level**					
**Place of residence**					< 0.001
Urban	3,827	66.8	60.3	39.7	
Rural	1907	33.2	40.9	59.1	
**Region**					< 0.001
Banjul	61	1.1	60.2	39.8	
Kanifing	1,047	18.3	60.9	39.1	
Brikama	2,353	41.0	69.3	30.7	
Mansakonko	244	4.2	48.3	51.7	
Kerewan	655	11.4	37.9	62.1	
Kuntaur	336	5.9	40.1	59.9	
Janjanbureh	361	6.3	47.7	52.3	
Basse	677	11.8	41.0	59.0	

Source: Computed from 2019–2020 Gambia Demographic and Health Survey (GDHS)

### Bivariate association between timely initiation of ANC services among reproductive age women and independent variables

The analysis as presented in Table 1 revealed that the overall prevalence of timely initiation of ANC services among reproductive age women in The Gambia was 43.0%. It was found that approximately 55% of the women aged 30–34 years initiated timely ANC services when pregnant. The timely initiation of ANC services was also phenomenal among married women (51.3%), women who were followers of Islam (50.3%), those who declared they do not watch television at all (57.1%) and those from the poorest homes (58.7%). Similarly, women residing in rural areas (59.1%), those who had given birth to more than four (52.5%), women who stated that distance to the health facility was a big problem (54.1%) as well as women from the Kerewan region sought ANC services on time.

### Determinants of timely initiation of ANC services among reproductive age women in The Gambia

Table 2 presents the fixed effects results of factors associated with timely initiation of ANC services. With women aged 15–19 years as reference, women aged 30–34 [aOR = 1.79, 95% CI = 1.30–2.47] have higher odds of seeking timely ANC services. Also, women who were married [aOR = 2.69, 95% CI = 1.85–3.90] had higher odds of seeking ANC services early than those who were not married. The findings also revealed that, women who had given birth to two children [aOR = 0.74, 95% CI = 0.6 -0.91] had lower odds of initiating timely ANC as compared to those who have given birth only once. Relative to women from the poorest households, women from the richest households [aOR = 1.63, 95% CI = 1.20-2.20] had higher odds of seeking timely ANC services. Women who reside in the rural areas [aOR = 1.72-95%CI = 1.34, 2.20] also had higher odds of seeking timely ANC services than those residing in the urban areas. The odds of seeking timely ANC was high in women living in the Kerewan region [aOR = 2.18, CI = 0.91-2.08] as compared to those in Banjul.

**Table 2:** Factors associated with timely initiation of ANC among reproductive age women in The Gambia using GDHS 2019–2020

Variable	Model O	Model I aOR [95% CI]	Model II aOR [95% CI]	Model III aOR [95% CI]
**Fixed effect results**				
**Individual level**				
**Age (years)**				
15–19		1 [1.00,1.00]		1 [1.00,1.00]
20–24		1.13 [0.86,1.49]		1.13 [0.86,1.48]
25–29		1.24 [0.92,1.66]		1.27 [0.95,1.70]
30–34		1.74^***^ [1.26, 2.40]		1.79^***^ [1.30, 2.47]
35–39		1.55^*^ [1.2, 2.31]		1.62^**^[1.16, 2.27]
40–44		1.47^*^ [1.01,2.13]		1.55^*^ [1.07,2.25]
45–49		1.31 [0.83, 2.07]		1.34 [0.85, 2.12]
**Marital status**				
Never married		1 [1.00,1.00]		1 [1.00,1.00]
Married		2.93^***^ [2.02, 4.25]		2.69^***^ [1.85, 3.90]
Widowed/Divorced/Separated		2.38^***^ [1.47, 3.86]		2.30^***^ [1.42, 3.73]
**Educational status**				
No education		1 [1.00,1.00]		1 [1.00,1.00]
Primary		1.06 [0.90, 1.24]		1.08 [0.93, 1.27]
Secondary		0.91 [0.78, 1.06]		0.96 [0.83, 1.12]
Higher		1.25 [0.88, 1.77]		1.26 [0.89, 1.80]
**Religion**				
Islam		1 [1.00,1.00]		1 [1.00,1.00]
Christianity		1.04 [0.63, 1.69]		1.18 [0.72, 1.91]
**Frequency of watching television**				
Not at all		1 [1.00,1.00]		1 [1.00,1.00]
Less than once a week		0.89 [0.75, 1.03]		0.93 [079, 1.09]
At least once a week		0.82^*^ [0.70, 0.96]		0.92 [0.77, 1.08]
**Parity**				
1 birth		1 [1.00,1.00]		1 [1.00,1.00]
2 births		0.73^**^ [0.60, 0.90]		0.74^**^ [0.60, 0.91]
3 births		0.89 [0.71, 1.10]		0.90 [0.72, 1.13]
4 or more births		0.76^*^ [0.61, 0.96]		0.76^*^ [0.60, 0.95]
**Getting medical help for self: distance to health facility**				
Big problem			1 [1.00,1.00]	1 [1.00,1.00]
Not a big problem			1.05 [0.92, 1.21]	1.05 [0.91, 1.20]
**Wealth index**				
Poorest			1 [1.00,1.00]	1 [1.00,1.00]
Poorer			0.92 [0.78, 1.10]	0.94 [0.75, 1.12]
Middle			0.98 [0.80, 1.21]	1.01 [0.81, 1.26]
Richer			1.09 [0.85, 1.39]	1.10 [0.84, 1.43]
Richest			1.65^***^ [1.25, 2.17]	1.63^**^ [1.20, 2.20]
**Getting medical help for self: distance to health facility**				
Big problem			1 [1.00,1.00]	1 [1.00,1.00]
Not a big problem			1.05 [0.92, 1.21]	1.05 [0.91, 1.20]
**Place of residence**				
Urban			1 [1.00,1.00]	1 [1.00,1.00]
Rural			1.71^***^ [1.34, 2.18]	1.72^***^ [1.34, 2.20]
**Region**				
Banjul			1 [1.00,1.00]	1 [1.00,1.00]
Kanifing			0.94 [0.67, 1.32]	0.95 [0.61, 1.34]
Brikama			0.67^*^ [0.48, 0.93]	0.66^*^ [0.47, 0.92]
Mansakonko			1.41 [0.94, 2.11]	1.38 [0.91, 2.08]
Kerewan			2.18^***^ [1.50, 3.19]	2.18^***^ [1.48, 3.21]
Kuntaur			1.76^**^ [1.18, 2.67]	1.71*[1.13, 2.59]
Janjanbureh			1.38 [0.93, 2.04]	1.35 [0.91, 2.02]
Basse			01.89^***^ [1.31, 2.72]	1.82^**^ [1.25, 2.64]
**Random effect model**				
PSU variance (95% CI)	0.46[0.35–0.60]	0.17[0.12–0.26]	0.43 [0.33–0.58]	0.18 [0.128–0.27]
ICC	0.1229385	0.0550171	0.1166237	0.0537193
Wald chi-square	Reference	194.03^***^	84.59^***^	255.63^***^
**Model fitness**				
Log-likelihood	-3832.6058	-3753.2352	-3786.991	-3713.5547
AIC	7669.212	7536.47	7611.982	7491.109
BIC	7682.52	7636.283	7738.411	7704.043

Exponential coefficients; 95% confidence intervals in brackets

1 = Reference category; *ICC* Intra-Class Correlation, *AIC* Akaike’s Information Criterion, *BIC* Bayesian Information Criterion, *PSU* Primary Sampling Unit

*aOR* adjusted odds ratios, *CI* Confidence Interval

^*^p < 0.05

^**^p < 0.01

^***^p < 0.001

### Random effect (measures of variation) results

The ICC value for the null model (0.1229385) depicts that 12.2% of the variation in timely ANC service utilization was attributed to the variation between clusters. This variation between clusters then reduced to 5.5% in Model I that is individual-level only model (ICC = 0.0550171). In the contextual level model (Model II), the ICC increased to 11.6% (ICC = 0.1166237), while in the last (Model III), it decreased to 5.3% (ICC = 0.0537193). This reiterates that the variations in the timely initiation of ANC are attributed to the individual and contextual level factors. The complete model (Model III) with individual and contextual level factors had the lowest Akaike Information Criterion (AIC) compared to the other models affirming the goodness of fit. Also, the final model showed the highest Log-likelihood (-3713.5547) value affirming the goodness of fit of the final model.

## Discussion

This study examined the prevalence and factors associated with timely initiation of ANC services during pregnancy among reproductive aged women in The Gambia. The WHO recommends a minimum of eight ANC contact with the first visit within 12 weeks of gestation [11]. Despite the recommendations by WHO and the numerous benefits of timely ANC, evidence shows the overall prevalence of timely ANC initiation in 36 SSA countries remains low [16]. The prevalence of timely initiation of ANC services among reproductive aged women in this study is 43%. This result is similar to other previous findings in Burkina Faso (43.4%) and Lesotho (43.3%) [16]. However, this finding is higher than what have been reported in several other studies conducted in LMICs including Ethiopia (27.5%) [12], (27.1) [23] (26.2%) [24], (17.4%) [25]; Benin (24.6%) [26], Tanzania (29%) [27] and Guinea (23%) [28]. However the prevalence of timely initiation of ANC services among reproductive age women in The Gambia is lower than the prevalence recorded globally (58.6%) [29] and in other studies from countries such as Ethiopia (46.8%) [30], (47.4%) [31]; Nepal (70%) [17], Ghana (57%) [13] and Cameroon (46%) [32]. The variations in the prevalence rate could be attributed to the fact that interventions implemented to improve maternal health services utilization in these countries might differ and also the variations might be attributed to the methodological design of the study and population [33].

This study found maternal age to have a significant association with the timely initiation of ANC services. Relative to women aged 15–19, those aged 30–34 and above had higher odds of initiating timely ANC services. This finding is in line with studies carried out in Ghana [13], Ethiopia [34], and Cameroon [32]. Most cultures and societies frown at teenage pregnancy and so teenage mothers feel ridiculed and stigmatized hence the more reason for their reluctance to initiate ANC services early in order to avoid shame, embarrassments and unfair treatment [35]. Furthermore, increasing maternal age might be associated with early pregnancy awareness [36]. Therefore younger women may be less likely to detect early pregnancy signs and might not initiate early ANC [37].

This study revealed that, reproductive aged women who had two births have lower odds of initiating ANC services on time as compared to those who had one. This finding is consistent with other studies which confirmed that women with high parity are noted to report late for ANC services during pregnancy [28, 32, 38]. This association could be linked to the fact that when women do not encounter any complications during their first gestation, they might be reluctant to utilize timely ANC s in their subsequent pregnancies [28, 39]. Also, some high-parity women might not have enough time to attend ANC services on time as they are occupied with the responsibilities of taking care of their children [40]. In addition, high parity women who were successful in their previous pregnancy might feel they know more about pregnancy, and its related issues and are conversant with what transpires during ANC hence their delay in initiating ANC services [35, 41]. Simkhada et al. [40] mentioned that if pregnant women do not receive treatment that meets their expectations during their previous ANC visits, they may not be willing to attend in their current pregnancies.

Consistent with existing literature, [38, 40, 42] we found that married women had higher odds of initiating timely ANC than those who were never married. Our finding might be attributed to the fact that married women are being supported by their partners and they have no feeling of shame in exposing their pregnancy hence they are bold in initiating timely ANC services [40].

It has also been revealed in our study that, women from the richest households have higher odds of seeking ANC services within the first 12 weeks of gestation compared to those from the poorest households. This finding is consistent with findings in Ghana [13], Nigeria [43], Nepal [17], and Cameroon [32] which revealed that women with high socioeconomic status have high tendencies to initiate timely ANC services during pregnancy relative to those who are in the poorest households. Albeit ANC services are provided free for all pregnant women in the Gambia [33], women from poor households may not be able to afford transportation costs and other indirect costs like household and work obligations in order to seek ANC [44, 45]. On the other hand, women from wealthier households are more likely to afford the direct cost (transportation costs) and meet their household and work obligations, in order to initiate timely ANC services. Another plausible reason could be that women from poor households lack financial capacity to support their daily living and, therefore might use most of the time for economic activities to provide for the family rather than their health.

Relative to women living in urban areas, those in rural areas had higher odds of timely initiation of ANC services within the first 12 weeks of gestation. This finding is counterintuitive to other studies from Ethiopia [46], Kenya [47], and Nigeria [48]. The public health system in The Gambia is made up of seven Divisional Health Teams (DHTs) dispersed around the nation, which provide both on-site clinic services and mobile maternity and child health clinics that go to remote areas. These initiatives in the rural areas might have contributed to higher initiation of ANC in these areas than the urban [49].

### Strengths and limitations of the study

This study employed data from the nationally representative GDHS and therefore findings could be generalized to the entire population of women in The Gambia. Again, another strength is the use of multilevel analysis to assess the factors associated with the timely initiation of ANC care services. Despite the study yielding significant results, we noted a couple of limitations. Causal inference could not be drawn due to the cross-sectional nature of the study. Also, the respondents in the GDHS survey relied on their memories to give self-reports and this could have led to recall bias. Another limitation for the study was that the new DHS methodology for multilevel analysis was not utilized but a robust multilevel analysis earlier recommended by DHS was applied.

## Conclusion

Despite the recommendations by the WHO for all pregnant women to initiate timely ANC services within the first 12 weeks of gestation, the prevalence of timely ANC initiation among reproductive age women in The Gambia was low. Factors such as maternal age, marital status, parity, wealth status, place of residence and religion were associated with timely initiation of ANC. The findings therefore suggests that interventions that are needed to improve timely initiation of ANC services should be focused on these factors. Teenage pregnant women and other unmarried pregnant women should be encouraged to initiate ANC on time without any feelings of shame. Efforts must be made to ensure equity for both the richest and the poorest in assessing timely ANC services since it will help in reducing the high rates of maternal and under-five mortalities recorded in The Gambia.

## Data Availability

The datasets used for this study is openly available and can be accessed via https://dhsprogram.com/data/dataset_admin/index.cfm.

## Abbreviations

AIC: Akaike’s Information Criterion
ANC: Antenatal care
aOR: Adjusted odds ratio
BIC: Bayesian information criterion
CIs: Confidence intervals
DHTs: Divisional Health Teams
EAs: Enumeration Areas
GBoS: Gambia Bureau of Statistics
GDHS: Gambia Demographic and Health Survey
LGAs: Local Government Areas
LMICs: Low and middle-income countries
MOH: Ministry of Health
SDGs: Sustainable Development Goals
SSA: Sub Saharan Africa
UNFPA: United Nations Population Fund
UNICEF: United Nations International Children’s Fund
VIF: Variance Inflation Factor
